# Gender differences in perceived food healthiness and food avoidance in a Swedish population-based survey: a cross sectional study

**DOI:** 10.1186/s12937-020-00659-0

**Published:** 2020-12-29

**Authors:** Linnea Bärebring, Maria Palmqvist, Anna Winkvist, Hanna Augustin

**Affiliations:** Department of Internal Medicine and Clinical Nutrition, Sahlgrenska Academy, University of Gothenburg, Box 459, 40530 Gothenburg, Sweden

**Keywords:** Health, Diet, Food avoidance, Beliefs

## Abstract

**Background:**

The aim of this work was to study potential gender differences in perceived food healthiness and food avoidance in a population-representative sample of the Swedish adult population.

**Methods:**

A questionnaire regarding diet and health was posted to 2000 randomly selected residents in Sweden, aged 20–65 years. Questions were posed regarding which foods or food components the participants avoided due to perceived unhealthiness and how healthy they believed the food items to be. The pre-specified food components included sugar, carbohydrate, gluten, lactose, dairy, fat, saturated fat, red meat, white flour, salt, alcohol and food additives (specifically glutamate, sweetening, preservative and coloring agents). Chi square tests were used to study differences in perceived food healthiness and food avoidance depending on gender.

**Results:**

Around 50% reported avoidance of sugar (51.6%) and sweeting agents (45.2%), whereas fewer reported avoidance of saturated fat (16.8%) and salt (10.6%). Women were more likely than men to avoid gluten (AOR [95% CI] 2.84 [1.33–6.05]), red meat (3.29 [1.86–5.80]), white flour (2.64 [1.65–4.21]), preservatives (1.7 [1.07–2.70]) and coloring agents (2.10 [1.29–3.41]) due to perceived unhealthiness. Gender differences were also apparent in perceived healthiness of sugar, gluten, dairy, red meat, white flour, alcohol and food additives, where women tended to be more negative than men in their attitudes. Women more often said to read new findings in media about diet (16% vs 9%, *p* = 0.029) and prioritize a healthy lifestyle (35% vs 25%, *p* = 0.015). More than a third of both women and men reported worrying over the healthiness of their diet, and a higher proportion of women than men (18% vs 11%, *p* = 0.015) agreed with the statement that they were often anxious over having an unhealthy diet.

**Conclusions:**

Women in this population-based study of residents in Sweden were more likely than men to avoid eating gluten, red meat, white flour and food additives due to perceived unhealthiness, and reported more diet and health related anxiety. Future research to identify effective ways of promoting healthy eating for both women and men, while minimizing diet-health related anxiety, is highly warranted.

**Supplementary Information:**

The online version contains supplementary material available at 10.1186/s12937-020-00659-0.

## Background

Diet is a major lifestyle determinant of health. Improving dietary habits by reducing intake of sodium, and increasing intake of whole grain and fruit could greatly decrease both morbidity and mortality from non-communicable disease [1]. Further, limiting intake of sugar and saturated fatty acids and increasing intake of fiber and unsaturated fatty acids are also beneficial for public health [2]. Women tend to report a healthier diet than men [3–5], which could in part explain why mortality rates are lower among women [6]. A national Swedish survey from 2011 showed that women reported higher intakes of fruit, berries, vegetables, water, tea, sweets and desserts. Men reported higher intakes of potatoes, bread, pasta, pizza, pie, red meat, sausage, coffee, soft drinks, lemonade, energy and sports drinks and alcohol [4].

As a diet in line with the current evidence based guidelines decreases risk of all-cause and cause-specific mortality [1, 2] – even more so among men than women, according to a Swedish study [5] –it is important to study the factors that determine dietary intake. A multi-national study including participants from 23 countries revealed that women place greater importance to healthy eating than do men, and that health beliefs explain a large proportion of dietary behavior [7]. Finnish research also demonstrated gender gaps in health information behavior, due to that women are more interested in and actively seek health-related information to a larger extent than do men. In addition, women are more attentive than men as to how the goods they purchase in everyday life affect their health [8]. As health believes in relation to food are likely contributors to dietary intake, more knowledge on gender differences in health believes and food avoidance could help facilitate public health initiatives to promote healthy eating in both women and men. However, little is known of gender differences in health beliefs or food avoidance for specific food items. The aim of this study was to compare perceived food healthiness and food avoidance among women and men, using a population-representative sample of residents in Sweden.

## Methods

### Study design

In January and February 2017, a questionnaire was sent via post to 2000 individuals. The study and the representativeness of the participants has been previously described [9]. The prospective participants were randomly selected from the Swedish Population Register that includes addresses of all persons who are registered as residents in Sweden. Participants from all parts of Sweden, between ages of 20–65 years, were eligible. This age range was chosen to primarily recruit participants with autonomy over their dietary intake. The exclusion criteria were classified personal information or residents who did not have a registered Swedish address. This study was approved by the Regional Ethics Committee in Gothenburg, Sweden. All participants were informed that returning the answered questionnaire was regarded as informed consent to participate in the study. All questionnaires were completely anonymous and data could not be traced back to the individual participants. Thus, no reminder was posted.

### Data collection

The questionnaire was six pages long, and took approximately 10–15 min to answer, and included questions on demographic variables, general health, perceived healthiness of foods, and statements regarding diet and health. The questionnaire was tested for clarity in a convenient sample of 10 participants in a wide age range, and only small adjustments to language and age categories were performed. Questionnaires with more than 20% missing data were excluded from the analyses. In addition, participants with non-binary gender identity were excluded from the current analyses.

Demographic data collected included gender, age, income, education and employment. Health data collected were self-reported weight and height, illness or food intolerance. Questions on the avoidance and perceived healthiness of specific foods or food components were posed for sugar, carbohydrates, fat, saturated fat, alcohol, red meat, dairy, white flour, salt, gluten, lactose and food additives (E-numbers, sweetening agents, preservative agents and coloring agents). These food components were chosen as they are commonly mentioned in terms of healthy eating, both in dietary recommendations and dietary fads (e.g. clean eating, anti-inflammatory diet etc.). Questions were formulated as *“Which of the following dietary components do you avoid, because you perceive it to be unhealthy?”* and *“What is your perception of the following dietary components?”.* Options were *very unhealthy, partly unhealthy, partly healthy, very healthy or no opinion*. Participants also were asked to agree or disagree with the following statements regarding health and diet: *I am interested in diet, I often read new findings on diet in the media, A healthy lifestyle is important to me, I worry that my diet is unhealthy and I often have anxiety over by diet being unhealthy*. The options provided were *agree completely, partly agree, partly disagree, disagree completely or no opinion*. This article conforms to the STROBE reporting format (Additional file 1).

### Statistical analyses

Median and quartiles 1–3 (Q1-Q3) and N (%) are used for continuous and categorical data, respectively. Gender differences in sociodemographic characteristics and in avoiding certain foods or food components were studied using chi square tests for categorical data, and Mann- Whitney U test for continuous variables. To control for potential confounding, a sensitivity analysis of food avoidance was also performed using multivariable logistic regression analysis (avoidance: no=0, yes=1), adjusted for age, education, income and employment. From these analysis, adjusted odds ratios (AOR) and 95% confidence intervals (95% CI) are presented. Gender differences in perceived healthiness of foods as well as agreement with claims related to diet and health were studied using chi square tests. The statistical software SPSS 22.0 (Armonk, New York: IBM Corp.) was used for all analyses. Significance was accepted at *p* < 0.05.

The sample size was based on recruiting a representative sample of the population, assuming 5% margin of error and 95% confidence interval, which would require 385 respondents. Assuming a 20% response rate, questionnaires were sent to 2000 individuals.

## Results

**Fig. 1:**
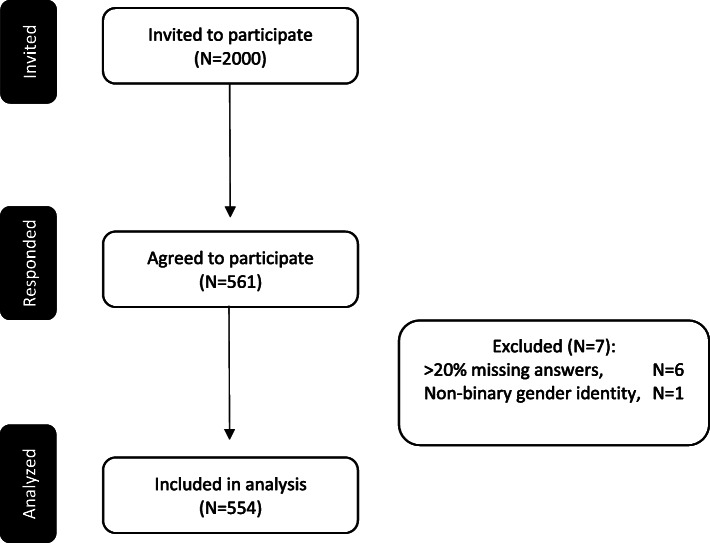
Flow chart of the study recruitment and data collection

A total of 561 questionnaires were answered, making the response rate 28% (Fig. 1). A total of 7 questionnaires were excluded due to exceeding the limit for missing data (*N* = 6) or non-binary gender identity (*N* = 1), and the total number of included participants was thus 554. A total of 55% of the included participants were women. A total of 17% (*N* = 90) reported to have a food allergy or intolerance, with no apparent gender differences (Table 1).

**Table 1:** Descriptive characteristics of the study participants

	All (***N*** = 554)	Females (***N*** = 306)	Males (***N*** = 248)	
	**Median (Q1-Q3)**	**Median (Q1-Q3)**	**Median (Q1-Q3)**	P*
**Weight (kg)**	75 (65–86)	68 (62–78)	83 (75–93)	< 0.001
**Height (cm)**	172 (167–180)	168 (163–171)	180 (176–185)	< 0.001
**BMI (kg/m**^**2**^**)**	24.8 (22.3–27.7)	24.2 (21.8–27.8)	25.1 (23.4–27.7)	0.006
	**N (%)**	**N (%)**	**N (%)**	P**
**Age (years)**				0.092
20–24	32 (6)	21 (7)	11 (4)	
25–30	39 (7)	26 (9)	13 (5)	
31–36	58 (11)	35 (11)	23 (9)	
37–45	100 (18)	55 (18)	45 (18)	
46–55	152 (27)	88 (29)	64 (26)	
56–66	171 (31)	80 (26)	91 (37)	
**Monthly income (€)**				0.001
< 1000	45 (8)	32 (11)	13 (5)	
1000–1500	42 (8)	26 (9)	16 (6)	
1500–2000	37 (7)	20 (7)	17 (7)	
2000–2500	55 (10)	33 (11)	22 (9)	
2500–3000	95 (17)	61 (20)	34 (14)	
3000–4000	158 (29)	86 (28)	72 (29)	
> 4000	119 (22)	46 (15)	73 (30)	
**Education**				0.018
Primary, ≤9 years	32 (6)	14 (5)	18 (7)	
Secondary, 2 years	69 (12)	35 (12)	34 (14)	
Secondary, 3 years	135 (25)	63 (21)	72 (30)	
Folk high school	17 (3)	11 (4)	6 (2)	
University, < 3 years	90 (16)	61 (20)	29 (12)	
University, ≥3 years	204 (37)	120 (39)	84 (35)	
**Employment**				< 0.001
Employed, full time	371 (67)	185 (60)	186 (75)	
Employed, part time	86 (16)	68 (22)	18 (7)	
Unemployed	16 (3)	7 (2)	9 (4)	
Parental leave	10 (2)	8 (3)	2 (1)	
Student	21 (4)	15 (5)	6 (2)	
Other	49 (9)	23 (8)	26 (11)	
**Food allergy or intolerance**				
Yes	90 (17)	54 (18)	36 (15)	0.351
No	452 (83)	247 (82)	205 (85)	

*Differences between females and males, using Mann Whitney U test, **Differences between females and males, using chi square test

### Perceived food healthiness

The proportions of participants who considered foods or food components to be unhealthy can be seen in Table 2. The most common food components viewed as “very unhealthy” were sugar (53%), sweetening agents (51%), coloring agents (43%), alcohol (41%), preservative agents (33%), saturated fat (29%) and white flour (26%). There were gender differences in perceived healthiness, as the women rated food components sugar, gluten, dairy, red meat, white flour, alcohol, and food additives, as less healthy than the men did.

**Table 2:** Gender differences in perceived healthiness of foods and food components in a Swedish population (*N* = 554)

	Very healthy N (%)	Somewhat healthy N (%)	Somewhat unhealthy N (%)	Very unhealthy N (%)	No opinion N (%)	P*
**Carbohydrate**						0.218
Female	16 (5)	131 (43)	131 (43)	10 (3)	16 (5)	
Male	20 (8)	115 (47)	84 (34)	11 (5)	16 (7)	
**Fat**						0.427
Female	35 (12)	143 (47)	97 (32)	18 (6)	10 (3)	
Male	19 (8)	128 (52)	74 (30)	20 (8)	7 (3)	
**Saturated fat**						0.809
Female	9 (3)	53 (18)	111 (37)	91 (30)	39 (13)	
Male	11 (5)	49 (20)	87 (35)	72 (29)	28 (11)	
**Alcohol**						0.001
Female	3 (1)	10 (3)	146 (48)	140 (46)	6 (2)	
Male	11 (4)	23 (9)	121 (49)	86 (35)	7 (3)	
**Sugar**						0.010
Female	2 (0.7)	10 (3)	107 (35)	184 (60)	3 (1)	
Male	1 (0.4)	13 (5)	120 (48)	112 (45)	2 (0.8)	
**Salt**						0.470
Female	3 (1)	56 (19)	201 (66)	34 (11)	9 (3)	
Male	4 (2)	47 (19)	161 (65)	22 (9)	14 (6)	
**Gluten**						0.023
Female	5 (2)	71 (23)	81 (27)	18 (6)	128 (42)	
Male	8 (3)	61 (25)	51 (21)	4 (2)	121 (49)	
**Lactose**						0.370
Female	11 (4)	76 (25)	77 (26)	8 (3)	128 (43)	
Male	4 (2)	71 (29)	53 (22)	5 (2)	114 (46)	
**Dairy**						0.019
Female	54 (18)	156 (52)	61 (20)	5 (2)	27 (9)	
Male	41 (17)	159 (64)	28 (11)	3 (1)	16 (7)	
**Red meat**						0.001
Female	8 (3)	75 (25)	148 (49)	51 (17)	22 (7)	
Male	14 (6)	85 (34)	116 (47)	16 (7)	16 (7)	
**White flour**						< 0.001
Female	3 (1)	27 (9)	169 (55)	93 (31)	13 (4)	
Male	5 (2)	56 (23)	110 (45)	48 (20)	27 (11)	
**Sweetening agents**						< 0.001
Female	1 (0.3)	5 (2)	87 (29)	182 (60)	30 (10)	
Male	3 (1)	15 (6)	79 (32)	103 (42)	47 (19)	
**Preservative agents**						< 0.001
Female	0 (0)	9 (3)	135 (44)	119 (39)	42 (14)	
Male	4 (2)	18 (7)	109 (44)	64 (26)	53 (21)	
**Coloring agents**						< 0.001
Female	0 (0)	4 (1)	104 (34)	158 (52)	39 (13)	
Male	4 (2)	11 (4)	96 (39)	81 (33)	56 (23)	

*Differences between women and men derived by chi square test

### Food avoidance

**Fig. 2:**
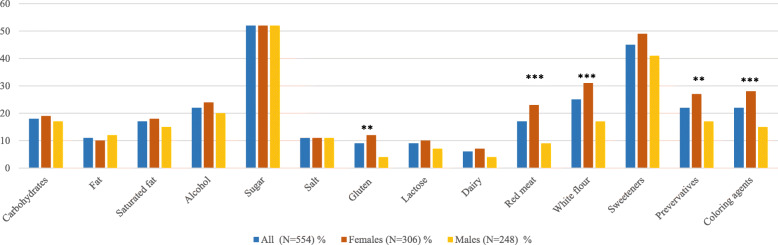
Reported avoidance of food or food components among all participants, and among women and men. Footnotes: **p < 0.01, ***p < 0.001

The proportion of reported avoidance of food or food components were as follows; 52% for sugar, 45% for sweetening agents, 25% for white flour, 22% for alcohol, 22% for preservative agents, 22% for coloring agents, 17% for saturated fat, 17% for red meat, 11% for fat, 11% for salt, 9% for gluten, 9% for lactose and 6% for dairy products (Fig. 2). When those with self-reported celiac disease, lactose intolerance or cow’s milk allergy were excluded from respective analysis, 7% still avoided gluten, 3% lactose and 5% dairy products. Women were two to three times more likely than men to avoid gluten (AOR [95% CI] 2.84 [1.33–6.05]), red meat (3.29 [1.86–5.80]), white flour (2.64 [1.65–4.21]), preservative (1.70 [1.07–2.70]) and coloring agents (2.10 [1.29–3.41]) due to perceived unhealthiness (Table 3). When those with self-reported celiac disease were excluded from respective analysis, the proportion who avoided gluten was still higher among women than men (9% vs 4%, *p* = 0.020).

**Table 3:** Gender differences in food avoidance due to perceived unhealthiness of pre-specified foods and food components

Avoids:	Males (***N*** = 248) AOR (reference)	Females (***N*** = 306) AOR (CI)	P*
Carbohydrates	1.0	1.34 (0.82–2.18)	0.238
Fat	1.0	0.68 (0.36–1.27)	0.222
Saturated fat	1.0	1.09 (0.65–1.83)	0.734
Alcohol	1.0	1.19 (0.75–1.90)	0.464
Sugar	1.0	1.01 (0.69–1.46)	0.977
Salt	1.0	0.95 (0.52–1.74)	0.858
Gluten	1.0	2.84 (1.33–6.05)	0.007
Lactose	1.0	1.43 (0.69–2.99)	0.339
Dairy	1.0	1.56 (0.63–3.88)	0.336
Red meat	1.0	3.29 (1.86–5.80)	< 0.001
White flour	1.0	2.64 (1.65–4.21)	< 0.001
Sweeteners	1.0	1.37 (0.94–2.00)	0.106
Preservatives	1.0	1.70 (1.07–2.70)	0.025
Coloring agents	1.0	2.10 (1.29–3.41)	0.003

*Difference between women and men, derived from logistic regression analysis. Adjusted for age, income, education and employment status. *AOR* Adjusted odds ratio, *CI* Confidence interval

### Interest and anxiety relating to diet and health

A higher proportion of women than men reported often reading findings regarding diet in the media (16% vs 9%, *p* = 0.029) and considered a healthy lifestyle to be important (35% vs 25%, *p* = 0.015). More than a third of both women and men reported worrying over the healthiness of their diet, but a higher proportion of women than men said they had anxiety over their diet being unhealthy (3% vs 0%, *p* = 0.015) (Table 4).

**Table 4:** Gender differences in attitudes to statements regarding food and health in a Swedish population

	Completely agree N (%)	Agree to an extent N (%)	Disagree to an extent N (%)	Totally disagree N (%)	No opinion N (%)	P*
**I am interested in diet**						0.088
Female	102 (33)	174 (57)	9 (3)	17 (6)	3 (1)	
Male	59 (24)	157 (63)	13 (5)	14 (6)	5 (2)	
**I often read new findings on diet in the media**						0.029
Female	48 (16)	160 (53)	39 (13)	44 (15)	13 (4)	
Male	23 (9)	119 (48)	48 (19)	48 (19)	10 (4)	
**A healthy lifestyle is important to me**						0.015
Female	107 (35)	162 (54)	19 (6)	8 (3)	6 (2)	
Male	63 (25)	160 (65)	17 (7)	8 (3)	0 (0)	
**I worry that my diet is unhealthy**						0.778
Female	10 (3)	103 (34)	53 (17)	130 (43)	8 (3)	
Male	9 (4)	81 (33)	53 (22)	96 (39)	7 (3)	
**I often have anxiety over my diet being unhealthy**						0.015
Female	10 (3)	46 (15)	48 (16)	197 (65)	3 (1)	
Male	0 (0)	28 (11)	34 (14)	181 (73)	5 (2)	

*Derived by chi square test

## Discussion

The results of this study show that there are gender differences in both perceived food healthiness and in food avoidance in Sweden. Overall, women reported more negative perceptions on the healthiness of sugar, gluten, dairy, red meat, white flour, alcohol and food additives. In addition, women were more likely to avoid gluten, red meat, white flour and food additives. Women also reported more anxiety related to food and health.

We found that there are gender differences in perceived healthiness of food that impacts dietary behavior. Previous studies show that women focus on nutritional value of food [10] and prioritize healthy eating [7] more so than do men. We found that the foods or food components most commonly viewed as “very unhealthy” and most commonly avoided were sugar, food additives, alcohol, saturated fat and white flour. This is in line with previous findings that women perceive sweet foods as less healthy [11] and avoid consumption of high fat foods to a higher extent [7], compared to men. A Swedish national survey from 2016 showed that women perceive the risk of falling ill through harmful substances such as chemicals in their diet, as higher than men [12]. This might, at least in part, explain why women had more negative views on food additives such as sweetening, coloring and conserving agents. Though all approved food additives are considered safe for human consumption, our findings suggest that there is a widespread concern of the health effects of these substances.

It is noteworthy that both women and men (but women more so than men) had more negative views on food additives than of established dietary risk factors such as salt, saturated fat and alcohol. This is possibly due to the recent year’s trend toward eating “clean” [13], which refers to consumption of unprocessed, whole foods and sometimes the elimination of entire food groups (e.g. dairy, sugar or gluten) [14]. Though perceived as healthy by many [14], “clean eating” does not guarantee a high quality diet [15] and could be associated with disordered eating [16]. As women’s dietary behavior to a greater extent than men’s seems impacted by perceived healthiness and is more likely to change over time [17] –dietary fads might have a greater impact on women’s diet. Previous findings from the current research project showed that women were indeed more likely than men to keep a specific diet and attempt to lose weight [9]. This could also be a reflection of women’s greater tendency to be impacted by dietary fads and trends. The specific diet, or foods or food components that are avoided likely differs over time, but this needs verification in longitudinal studies.

The observed gender differences in the current and previous studies might have significant implications for public health. Findings are consistent that women are more health conscious than men –both in general [8] and in specific regards to their diet [7]. This might have parallel effects, where women eat healthier than men but also have more body shape concerns and diet-related anxiety. Perceived diet-related risks are assessed by both emotional and cognitive considerations, among both women and men [18]. Thereby, simply providing more information on diet and health is unlikely to eliminate gender differences in food perception and avoidance. More research is needed to identify effective ways of promoting healthy eating for both women and men, while minimizing diet-health related anxiety.

### Strengths and limitation

Strengths of the current study include the relatively high response rate (28%) for this kind of study, and that the study sample is deemed population-representative. We have previously concluded that the sample seems representative of the general Swedish population in regards to prevalence of overweight and income, whereas the education level was slightly higher than the general Swedish population [9]. The proportions of women and men in the study are 55 and 45%, which indicated that women are slightly overrepresented (as the national gender distribution is 50% women, 50% men). Thus, there are small differences in sociodemographic data in this study sample, compared to the general population. The study results are thereby likely generalizable to the Swedish population in the current age span. Limitations include a lack of detailed dietary intake data to verify that reported food avoidance was also reflected in actual diet. In addition, a number of statistical tests were performed on several variables in this paper, and *p*-values should thereby be interpreted with caution. An additional limitation is the pre-specified answers that may have restricted the range of possible responses to the questions. Even though free text options were available, these were not frequently used. Future studies should consider combining quantitative and qualitative approaches to provide further clarity to the motivations for women’s more frequent food avoidance.

## Conclusions

Women in this population-based study of residents in Sweden were more likely than men to avoid eating gluten, red meat, white flour and food additives due to perceived unhealthiness, and reported more diet and health related anxiety. Future research to identify effective ways of promoting healthy eating for both women and men, while minimizing diet-health related anxiety, is highly warranted.

## Supplementary Information

**Additional file 1: **STROBE Statement—Checklist of items that should be included in reports of *cross-sectional studies.*

## Data Availability

Data cannot be made freely available as they are subject to secrecy in accordance with the Swedish Public Access to Information and Secrecy Act (Offentlighets- och sekretesslagen, OSL, 2009:400), but can be made available to researchers upon request (subject to a review of secrecy). Requests for data should be made to Linnea Bärebring (linnea.barebring@gu.se).
